# Risk of intracranial hemorrhage (RICH) in users of oral antithrombotic drugs: Nationwide pharmacoepidemiological study

**DOI:** 10.1371/journal.pone.0202575

**Published:** 2018-08-23

**Authors:** Sasha Gulati, Ole Solheim, Sven M. Carlsen, Lise R. Øie, Heidi Jensberg, Agnete M. Gulati, Mattis A. Madsbu, Charalampis Giannadakis, Asgeir S. Jakola, Øyvind Salvesen

**Affiliations:** 1 Department of Neurosurgery, St. Olavs University Hospital, Trondheim, Norway; 2 Department of Neuroscience, Norwegian University of Science and Technology (NTNU), Trondheim, Norway; 3 National Advisory Unit on Spinal Surgery, St. Olavs University Hospital, Trondheim, Norway; 4 National Advisory Unit on Ultrasound and Image-Guided Therapy, St. Olavs University Hospital, Trondheim, Norway; 5 Department of Endocrinology, St. Olavs University Hospital, Trondheim, Norway; 6 Department of Cancer Research and Molecular Medicine, Norwegian University of Science and Technology (NTNU), Trondheim, Norway; 7 Department of Neurology, St. Olavs University Hospital, Trondheim, Norway; 8 The Norwegian Patient Registry, Trondheim, Norway; 9 Department of Rheumatology, St. Olavs University Hospital, Trondheim, Norway; 10 Department of Neurosurgery, Sahlgrenska University Hospital, Gothenburg, Sweden; 11 Institute of Neuroscience and Physiology, University of Gothenburg, Sahlgrenska Academy, Gothenburg, Sweden; 12 Department of Public Health and General Practice, Norwegian University of Science and Technology (NTNU), Trondheim, Norway; Monash University, AUSTRALIA

## Abstract

**Background:**

The risks of intracranial haemorrhage (ICH) associated with antithrombotic drugs outside clinical trials are gaining increased attention. The aim of this nationwide study was to investigate the risk of ICH requiring hospital admission in users of antithrombotic drugs.

**Methods and findings:**

Data from the Norwegian Patient Registry and Norwegian Prescription Database were linked on an individual level. The primary outcome was incidence rates of ICH associated with use of antithrombotic drugs. Secondary endpoints were risk of ICH and fatal outcome following ICH assessed by Cox models. Among 3,131,270 individuals ≥18 years old observed from 2008 through 2014, there were 729,818 users of antithrombotic medications and 22,111 ICH hospitalizations. Annual crude ICH rates per 100 person-years were 0.076 (95% CI, 0.075–0.077) in non-users and 0.30 (95% CI, 0.30–0.31) in users of antithrombotic medication, with the highest age and sex adjusted rates observed for aspirin-dipyridamole plus clopidogrel (0.44; 95% CI, 0.19–0.69), rivaroxaban plus aspirin (0.36; 95% CI, 0.16–0.56), warfarin plus aspirin (0.34; 95% CI, 0.26–0.43), and warfarin plus aspirin and clopidogrel (0.33; 95% CI, 0.073–0.60). With no antithrombotic medication as reference, the highest adjusted hazard ratios (HR) for ICH were observed for aspirin-dypiridamole plus clopidogrel (6.29; 95% CI 3.71–10.7), warfarin plus aspirin and clopidogrel (4.38; 95% CI 2.71–7.09), rivaroxaban plus aspirin (3.82; 95% CI, 2.46–5.95), and warfarin plus aspirin (3.40; 95% CI, 2.99–3.86). All antithrombotic medication regimens were associated with an increased risk of ICH, except dabigatran monotherapy (HR 1.20; 95% CI, 0.88–1.65) and dabigatran plus aspirin (HR 1.79; 95% CI, 0.96–3.34). Fatal outcome within 90 days was more common in users (2,603 of 8,055) than non-users (3,228 of 14,056) of antithrombotic medication (32.3% vs 23.0%, p<0.001), and was associated with use of warfarin plus aspirin and clopidogrel (HR 2.89; 95% CI, 1.49–5.60), warfarin plus aspirin (HR 1.37; 95% CI, 1.11–1.68), aspirin plus clopidogrel (HR 1.30; 95% CI, 1.05–1.61), and warfarin (HR 1.19; 95% CI, 1.09–1.31). Increased one-year mortality was observed in users of antithrombotic medication following hemorrhagic stroke, subdural hemorrhage, subarachnoid hemorrhage, and traumatic ICH (all p<0.001). Limitations include those inherent to observational studies including the inability to make causal inferences, certain assumptions regarding drug exposure, and the possibility of residual confounding.

**Conclusions:**

The real-world incidence rates and risks of ICH were generally higher than reported in randomized controlled trials. There is still major room for improvement in terms of antithrombotic medication safety (clinicaltrials.gov NCT02481011).

## Introduction

Among hemorrhagic complications of antithrombotic medications, intracranial hemorrhage (ICH) may have particularly devastating consequences with high morbidity and mortality rates [1, 2]. Although a certain adverse risk for bleedings may be acceptable in the context of even greater protection against life-threatening ischemic events, it is important to quantify the magnitude of bleeding risk. Antithrombotic medications are generally assessed in randomized controlled trials (RCT), but included patients may not be representative of users in everyday clinical practice in terms of follow-up routines, age, gender, comorbidity, drug compliance, physical activity and polypharmacy [3]. Complications are rarely primary endpoints in RCTs and statistical power to evaluate complication rates may be limited since treatment periods are often shorter than in routine management of chronic conditions. Drifts in indications and treatment criteria may be seen in everyday practice and drug discontinuation due to precautionary concerns may be forgotten. Collectively, these factors may lead to other and potentially higher ICH rates in general clinical use than reported in RCTs. As a result, the risks of ICH associated with antithrombotic drugs outside clinical trials are gaining increased attention [2, 4–7]. The objective of this nationwide study was to investigate the risk of ICH and fatal outcome following ICH in users of antithrombotic medications.

## Methods

This nationwide study was conducted and reported with fidelity to the published protocol (S1 Protocol) [8]. Reporting is consistent with the strengthening the reporting of observational studies in epidemiology statement (S1 Checklist). Ethical approval and waiver of the requirement for obtaining patient consent were granted by the Regional Committee for Medical Research (2014/958).

### Study population

The study was conducted within the 4.7 million inhabitants (2008 Census) of Norway from January 1^st^, 2008, to December 31^st^, 2014. Two administrative registries were linked on an individual level by a unique 11-digit personal identifier. The registries linked were: (1) the Norwegian Patient Registry (NPR), which contains information about all admissions to Norwegian hospitals since 2008 with diagnoses coded according to the 10^th^ revision of the International Classification of Diseases (ICD-10) and (2) The Norwegian Prescription Database (NorPD), which contains information about all prescriptions dispensed in Norway since 2004 including type of drug according to the Anatomical Therapeutic Chemical (ATC) classification system, number of Defined Daily Doses (DDD), date of dispensing, quantity dispensed, and drug strength. The National Registry, a civil registration registry, provides vital statistics to NPR and NorPD. All residents ≥18 years during the study period included in NPR and/or NorPD were eligible for inclusion. Data from NPR and NorPD were de-identified before provided to the study authors.

### Outcome measures

The primary outcome was incidence rates of ICH associated with use of antithrombotic drugs. Both crude rates and age and sex adjusted rates were determined for the primary outcome. Secondary endpoints were risk of ICH and fatal outcome following ICH according to antithrombotic medication exposure assessed by Cox models. Adjustments for age, sex, concomitant medications, and comorbidity were performed for secondary outcomes.

### The Norwegian health care system

Acute illness requiring hospital admission is treated free of cost by the public health care system and insurance policies do not influence the management of ICH. Only public hospitals provide health care to patients with ICH. The health authorities cover all inpatient treatment of ICH. Except for a maximum annual deductible of 2,185 Norwegian Kroner (270 USD, 2014), prescription drugs are provided to patients without further costs.

### The Norwegian Prescription Database

Antithrombotic medications are only available at state regulated pharmacies and only dispensed to patients with a prescription from a physician. Pharmacies in Norway are required to register each drug dispensing in NorPD, ensuring complete registration. Diagnoses are registered for medications with reimbursement according to ICD-10 or version 2 of the International Classification of Primary Care. All filled prescriptions for oral formulations of antithrombotic medications were recorded including aspirin, dipyridamole, aspirin-dipyridamole, clopidogrel, prasugrel, ticagrelor, ticlopidine, warfarin, dabigatran, apixaban, rivaroxaban, dicumarol, and phenylindandion. Aspirin in limited packages is available over-the-counter without a prescription in Norway as an analgesic. However, over-the-counter turnover of aspirin is limited in Norway, and according to the Norwegian Institute of Public Health >98% of over-the-counter medication turnover is made up by ibuprofen, acetaminophen, nicotine, xylometazoline, xylometazoline-ipratropiumbromide, and cetirizine [9]. Dispensed prescriptions for renin-angiotensin system inhibitors, antiarrhythmic drugs, non-steroidal anti-inflammatory drugs, antidepressants, and proton pump inhibitors were considered concomitant medication.

### The Norwegian Patient Registry

NPR automatically receives information regarding diagnoses when patients receive inpatient treatment by public specialist health care services. Sensitivity, specificity, and positive predictive values for stroke diagnoses, including ICH, in NPR are found to be high, supporting that the registry is adequately complete and correct to provide data for epidemiological studies [10, 11]. NPR identified patients with a primary diagnosis of ICH, (ICD-10: I60-I62.0 and S06.3-S06.6) requiring hospitalization. ICH was classified as hemorrhagic stroke, non-traumatic or low-energy subdural hemorrhage, subarachnoid hemorrhage, or traumatic intracranial hemorrhage. If diagnoses of trauma or accident were present for the same hospital admission, the ICH event was classified as traumatic. Distinction between chronic subdural hemorrhage and acute subdural hemorrhage was not possible based on ICD-10 codes [12].

### Assessment of exposure to antithrombotic medications

Patients were followed until death or end of study period. Drug exposure and comorbidity were registered from January 1^st^ 2008 until outcome date (i.e. what came first of either the first ICH event or end of study period). Individual exposure periods for antithrombotic medications were calculated using DDD as a measuring unit [13]. Exposure was defined as having occurred when patients had drugs available and discontinuation as when they had no more drugs available. For each prescription an assumption was made that the patient was exposed from the date the drug was dispensed and for a number of days corresponding to the quantity of drug dispensed measured in DDD. We treated use of drugs in the analyses as time varying exposures, and patients could change exposure group according to dispensed prescriptions during the entire span of the study period. We calculated risk time (person-years) only for the active treatment period. Simultaneous exposure to >1 anticoagulant was considered to represent a phase where the patient was presumably switched from one anticoagulant to another; in such rare cases we therefore regarded the most recently dispensed anticoagulant as current exposure.

### Potential confounders and risk factors

The entire study population was screened for the following comorbidities: hypertension, atrial fibrillation, congestive heart failure, heart valve disease, thromboembolism (including ischemic stroke), vascular disease, diabetes mellitus, peptic ulcer, liver disease, alcohol abuse, osteoarthritis, extracranial bleeding, and chronic renal failure (Method A in S1 Appendix). Comorbidities were registered as dichotomous variables during the observation period, and not as time varying exposures. Apart from stated comorbid conditions, we also appraised use of certain medications (Table A in S1 Appendix), as they might influence risk of ICH, provide a better description of the population under investigation, and allow for future comparisons across studies.

### Statistics

Statistical analyses were performed with SPSS for Mac version 21.0 (IBM Corp., Chicago, IL), MySQL (Oracle), or R Statistical Software version 3.1. Due to multiple comparisons the statistical significance level was defined as P≤0.001. Age and sex adjusted incidence rates for the different drug exposures were computed using the method of direct standardization with the complete study population provided by NPR and NorPD as the reference population. Direct standardization was used to reduce the effect of potential confounders (i.e. age and sex distribution) that differ between the populations. Hazard ratios (HR) with 95% confidence intervals (CI) for ICH were estimated using Cox regression models with adjustments for age, sex, concomitant drugs, and comorbidity. The time variable in the Cox model was patient age. Case fatality was investigated at 90 days in addition to an analysis of overall survival until 12 months following ICH. For all outcome measures the statistician was blinded to drug exposure. Outcomes are presented only for antithrombotic regimens with ≥5000 users during the study period.

### Missing data

NPR and NorPD did not include individuals who never had any contact with public specialist health care services nor claimed any prescription during the study period. The number, age, and sex of unidentified, presumably healthy individuals were retrieved from Statistics Norway and imputed in post-hoc analyses on patient-years at risk, incidence rates of ICH, and adjusted HRs for ICH by drug exposure group.

### Protocol deviation

Traumatic intracranial hemorrhages were originally excluded in the study protocol, but were included in the study to better assess the role of antithrombotic medications in ICH and its potential association with physical damage following trauma. Moreover, it can be difficult to distinguish traumatic and non-traumatic ICH in a nationwide registry-based setting.

## Results

### Study population

Among 3,131,270 individuals observed, there were 729,818 users of antithrombotic medications and 22,111 ICH hospital admissions, including 8,665 with hemorrhagic stroke, 4,487 with subdural hematoma, 2,680 with subarachnoid hemorrhage, and 6,279 with traumatic intracranial hemorrhage (Figure A in S1 Appendix). The characteristics of patients by antithrombotic medication exposure at time of drug dispensing are presented in Table A in S1 Appendix.

### Primary outcome

**Fig 1:**
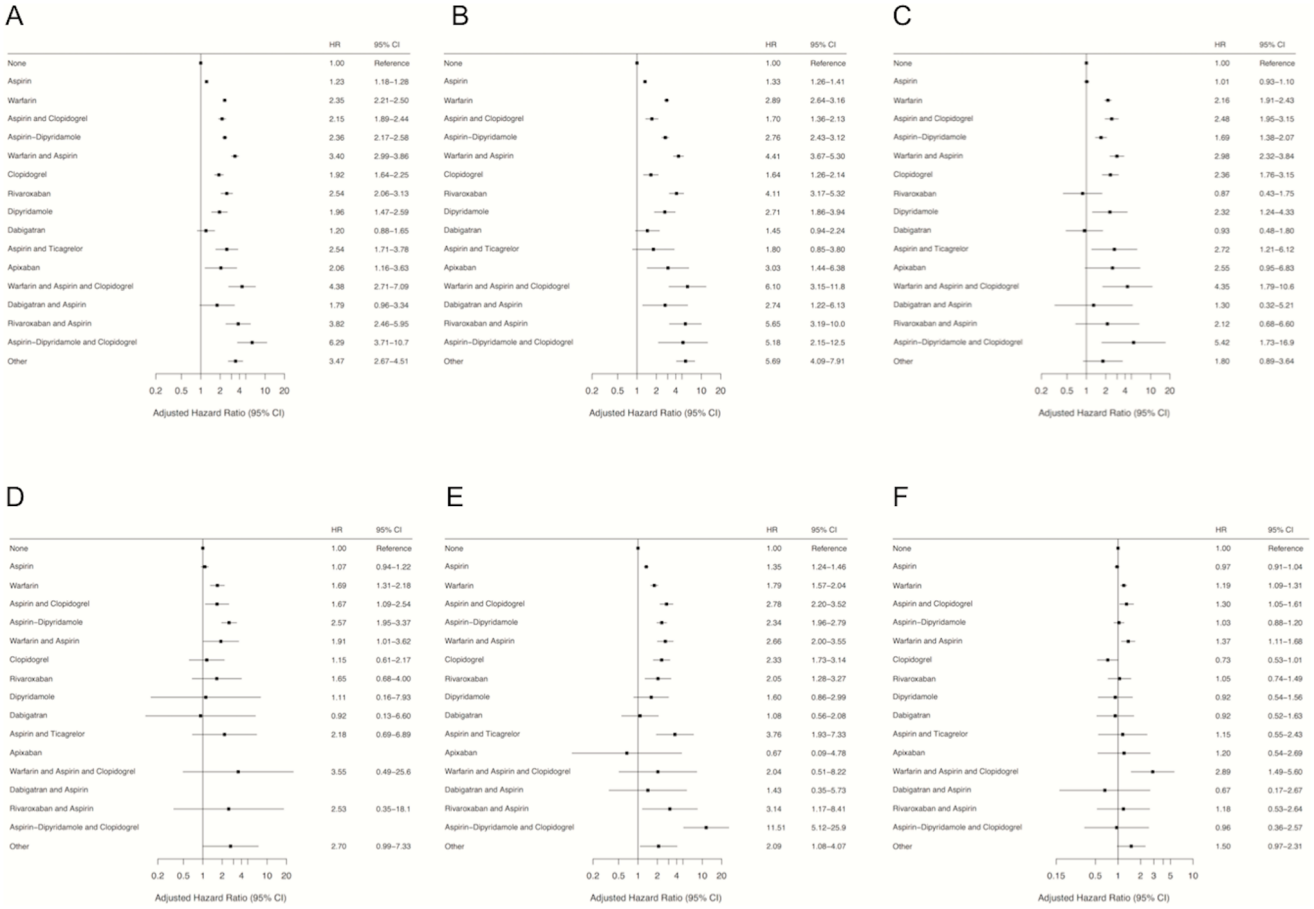
Impact of antithrombotic medication on risk of intracranial hemorrhage and fatal intracranial hemorrhage. Drug exposure was associated with increased risk for all outcomes (p<0.001). All HRs are adjusted for age, sex, comorbidity, and concomitant medication. (A) Risk of any intracranial hemorrhage. (B) Risk of hemorrhagic stroke. (C) Risk of subdural hemorrhage. (D) Risk of subarachnoid hemorrhage. (E) Risk of traumatic intracranial hemorrhage. (F) Risk of fatal outcome following intracranial hemorrhage.

The crude ICH rates per 100 person-years were 0.076 (95% CI, 0.075–0.077) in non-users and 0.30 (95% CI, 0.30–0.31%) in users of antithrombotic medications, with the highest rates observed for aspirin-dipyridamole plus clopidogrel (1.13; 95% CI 0.71–2.19), rivaroxaban plus aspirin (0.90; 95% CI 0.55–1.40), warfarin plus aspirin and clopidogrel (0.85; 95% CI, 0.49–1.36), and warfarin plus aspirin (0.75; 95% CI, 0.66–0.85). The highest age and sex adjusted rates ICH rates per 100 person-years were observed for aspirin-dipyridamole plus clopidogrel (0.44; 95% CI, 0.19–0.69), rivaroxaban plus aspirin (0.36; 95% CI, 0.16–0.56), warfarin plus aspirin (0.34; 95% CI, 0.26–0.43), and warfarin plus aspirin and clopidogrel (0.33; 95% CI, 0.073–0.60). Table 1 shows the patient-years at risk, crude incidence rates, and sex- and age-adjusted annual incidence rates for ICH by drug exposure group. As seen in Fig 1A, all antithrombotic medication regimens were associated with an increased risk of ICH, apart from dabigatran monotherapy and dabigatran plus aspirin. With no antithrombotic medication as reference, the highest adjusted hazard ratios (HR) for ICH were observed for aspirin-dypiridamole plus clopidogrel (6.29; 95% CI 3.71–10.7), warfarin plus aspirin and clopidogrel (4.38; 95% CI 2.71–7.09), rivaroxaban plus aspirin (3.82; 95% CI, 2.46–5.95), and warfarin plus aspirin (3.40; 95% CI, 2.99–3.86). Incidence rates and adjusted hazard ratios for hemorrhagic stroke, subdural hematoma, subarachnoid hemorrhage, and traumatic intracranial hemorrhage according to antithrombotic medication exposure are presented in Table 2 and Fig 1B–1E, respectively.

**Table 1:** Patient-years at risk and incidence rates for ICH by drug exposure group.

Antithrombotic treatment	Patients, No.	Patient-years at risk	Events, No.	Crude Rate, Events per 100 Person-Years (95% CI)	Age and sex adjusted incidence rate, Events per 100 Person-Years (95% CI)
None	3108394	18498589.5	14056	0.076 (0.075–0.077)	0.093 (0.091–0.094)
Aspirin	594761	1998423.3	4701	0.24 (0.23–0.24)	0.12 (0.11–0.14)
Warfarin	151966	306658.9	1678	0.55 (0.52–0.57)	0.28 (0.24–0.31)
Aspirin plus Clopidogrel	83593	72635.1	264	0.36 (0.32–0.41)	0.20 (0.15–0.24)
Aspirin-Dipyridamole	59698	131792.9	650	0.49 (0.46–0.53)	0.30 (0.19–0.40)
Warfarin plus Aspirin	54152	35035.2	263	0.75 (0.66–0.85)	0.34 (0.26–0.43)
Clopidogrel	44771	43010.8	162	0.38 (0.32–0.44)	0.25 (0.16–0.34)
Rivaroxaban	23532	17873.3	91	0.51 (0.41–0.63)	0.26 (0.18–0.34)
Dipyridamole	22329	10868.0	49	0.45 (0.33–0.60)	0.15 (0.098–0.21)
Dabigatran	18541	16176.3	40	0.25 (0.18–0.34)	0.010 (0.057–0.13)
Ticagrelor plus Aspirin	11896	8829.8	25	0.28 (0.18–0.42)	0.20 (0.098–0.31)
Apixaban	8202	2688.0	12	0.45 (0.23–0.78)	0.17 (0.065–0.27)
Warfarin plus Aspirin and Clopidogrel	7682	2008.7	17	0.85 (0.49–1.36)	0.33 (0.073–0.60)
Dabigatran plus Aspirin	6929	2487.3	10	0.40 (0.19–0.74)	0.24 (0.028–0.45)
Rivaroxaban plus Aspirin	6740	2211.9	20	0.90 (0.55–1.40)	0.36 (0.16–0.56)
Aspirin-Dipyridamole plus Clopidogrel	5835	1072.6	14	1.31 (0.71–2.19)	0.44 (0.19–0.69)
Other antithrombotic medication	24734	9986.2	59	0.59 (0.45–0.76)	0.25 (0.18–0.32)

Patients could have multiple treatment courses with one drug or with different drugs.

**Table 2:** Incidence rates for hemorrhagic stroke, subdural hemorrhage, subarachnoid hemorrhage and traumatic intracranial hemorrhage by drug exposure group.

Antithrombotic treatment	Hemorrhagic stroke			Subdural hemorrhage			Subarachnoid hemorrhage			Traumatic intracranial hemorrhage		
	Events, No.	Crude Rate, Events per 100 Person-Years (95% CI)	Age and sex adjusted incidence rate, Events per 100 Person-Years (95% CI)	Events, No.	Crude Rate, Events per 100 Person-Years (95% CI)	Age and sex adjusted incidence rate, Events per 100 Person-Years (95% CI)	Events, No.	Crude Rate, Events per 100 Person-Years (95% CI)	Age and sex adjusted incidence rate, Events per 100 Person-Years (95% CI)	Events, No.	Crude Rate, Events per 100 Person-Years (95% CI	Age and sex adjusted incidence rate, Events per 100 Person-Years (95% CI)
None	4967	0.027 (0.026–0.028)	0.034 (0.034–0.035)	2701	0.015 (0.014–0.015)	0.019 (0.019–0.020)	2076	0.011 (0.011–0.012)	0.012 (0.011–0.012)	4312	0.023 (0.023–0.024)	0.027 (0.026–0.028)
Aspirin	2103	0.11 (0.10–0.11)	0.053 (0.040–0.066)	967	0.048 (0.045–0.052)	0.021 (0.018–0.025)	399	0.020 (0.018–0.022)	0.015 (0.011–0.018)	1232	0.062 (0.058–0.065)	0.033 (0.027–0.040)
Warfarin	813	0.27 (0.25–0.28)	0.12 (0.099–0.15)	454	0.15 (0.13–0.16)	0.076 (0.058–0.093)	77	0.025 (0.020–0.031)	0.024 (0.013–0.034)	334	0.11 (0.098–0.12)	0.051 (0.041–0.061)
Aspirin plus Clopidogrel	83	0.11 (0.09–0.14)	0.049 (0.036–0.062)	77	0.11 (0.084–0.13)	0.069 (0.034–0.10)	24	0.033 (0.021–0.049)	0.019 (0.0096–0.028)	80	0.11 (0.087–0.14)	0.060 (0.043–0.076)
Aspirin-Dipyridamole	317	0.24 (0.21–0.27)	0.13 (0.088–0.18)	112	0.085 (0.070–0.10)	0.029 (0.023–0.036)	68	0.052 (0.040–0.065)	0.033 (0.019–0.047)	153	0.12 (0.098–0.14)	0.099 (0.0050–0.19)
Warfarin plus Aspirin	132	0.38 (0.32–0.45)	0.19 (0.12–0.25)	69	0.20 (0.15–0.25)	0.096 (0.051–0.14)	10	0.029 (0.014–0.052)	0.011 (0.0030–0.020)	52	0.15 (0.11–0.19)	0.049 (0.032–0.067)
Clopidogrel	57	0.13 (0.10–0.17)	0.085 (0.052–0.12)	49	0.11 (0.084–0.15)	0.083 (0.0050–0.16)	10	0.023 (0.011–0.043)	0.011 (0.0035–0.018)	46	0.11 (0.078–0.14)	0.069 (0.038–0.10)
Rivaroxaban	60	0.34 (0.26–0.43)	0.16 (0.11–0.21)	8	0.045 (0.019–0.088)	0.024 (0.0021–0.045)	5	0.028 (0.009–0.065)	0.012 (0.00038–0.023)	18	0.10 (0.060–0.16)	0.065 (0.015–0.12)
Dipyridamole	28	0.26 (0.17–0.37)	0.083 (0.046–0.12)	10	0.092 (0.044–0.17)	0.028 (0.0099–0.046)	1	0.0092 (0.00023–0.051)	0.0024 (-0.0023–0.0070)	10	0.092 (0.044–0.17)	0.040 (0.0033–0.077)
Dabigatran	21	0.13 (0.08–0.20)	0.044 (0.024–0.063)	9	0.056 (0.025–0.11)	0.022 (0.0062–0.039)	1	0.0062 (0.00016–0.034)	0.0017 (-0.0016–0.0050)	9	0.056 (0.025–0.11)	0.027 (-0.0013–0.056)
Ticagrelor plus Aspirin	7	0.079 (0.03–0.16)	0.062 (-0.000094–0.12)	6	0.068 (0.025–0.15)	0.059 (-0.0012–0.12)	3	0.034 (0.0070–0.099)	0.016 (-0.0036–0.034)	9	0.10 (0.047–0.19)	0.066 (0.010–0.12)
Apixaban	7	0.26 (0.10–0.54)	0.10 (0.022–0.18)	4	0.15 (0.041–0.38)	0.051 (-0.0038–0.11)	0	0.00 (0.00–0.14)	0.00 (0.00–0.00)	1	0.037 (0.00094–0.21)	0.012 (-0.011–0.035)
Warfarin plus Aspirin and Clopidogrel	9	0.45 (0.20–0.85)	0.26 (0.00077–0.51)	5	0.25 (0.081–0.58)	0.044 (0.0039–0.085)	1	0.050 (0.0013–0.28)	0.012 (-0.012–0.036)	2	0.10 (0.012–0.36)	0.022 (-0.0086–0.052)
Dabigatran plus Aspirin	6	0.24 (0.09–0.53)	0.13 (-0.034–0.29)	2	0.08 (0.0097–0.29)	0.054 (-0.040 to 0.15)	0	0.00 (0.00–0.15)	0.00 (0.00–0.00)	2	0.080 (0.0097–0.29)	0.055 (-0.039–0.15)
Rivaroxaban plus Aspirin	12	0.54 (0.28–0.95)	0.25 (0.069–0.43)	3	0.14 (0.028–0.40)	0.030 (-0.0051–0.065)	1	0.045 (0.0011–0.25)	0.021 (-0020- 0.062)	4	0.18 (0.049–0.46)	0.056 (-0.0046–0.12)
Aspirin-Dipyridamole plus Clopidogrel	5	0.47 (0.15–1.09)	0.19 (0.0071–0.37)	3	0.28 (0.058–0.82)	0.064 (-0.0093–0.14)	0	0.00 (0.00–0.34)	0.00 (0.00–0.00)	6	0.56 (0.21–1.22)	0.19 (0.034–0.34)
Other antithrombotic medication	15	0.38 (0.27–0.52)	0.16 (0.10–0.21)	8	0.080 (0.035–0.16)	0.029 (0.0079–0.050)	4	0.040 (0.011–0.10)	0.024 (0.052–2.43)	9	0.090 (0.041–0.17)	0.040 (0.0084–0.072)

Patients could have multiple treatment courses with one drug or with different drugs.

### Secondary outcome

**Fig 2:**
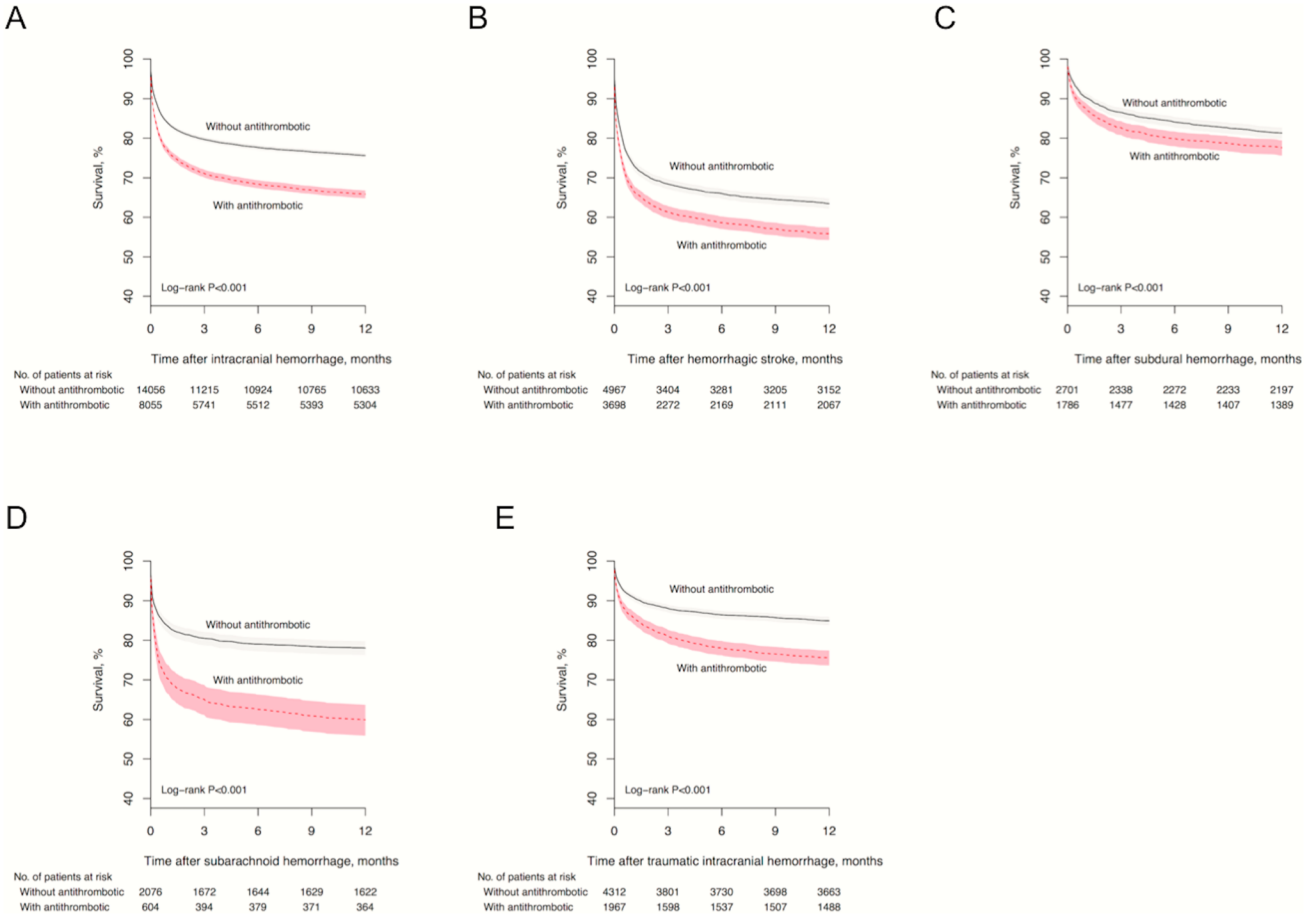
Survival following ICH in users and non-users of antithrombotic medications. There were significant differences in survival following ICH and in all four subgroups hemorrhagic stroke, subdural hemorrhage, subarachnoid hemorrhage, and traumatic intracranial hemorrhage between users and non-users of antithrombotic medications (all p<0.001). The shaded areas indicate 95% CIs. (A) Survival following any intracranial hemorrhage. (B) Survival following hemorrhagic stroke. (C) Survival following subdural hemorrhage. (D) Survival following subarachnoid hemorrhage. (E) Survival following traumatic intracranial hemorrhage.

As presented in Fig 2, one-year mortality following ICH was higher in users of antithrombotic medications than non-users in the subgroups hemorrhagic stroke, subdural hematoma, subarachnoid hemorrhage, and traumatic intracranial hemorrhage (all p<0.001). Death within 90 days was more common in users (2,603 of 8,055) than non-users (3,228 of 14,056) of antithrombotic medication (32.3% vs 23.0%, p<0.001). The proportions of patients with fatal outcome within 90 days following ICH according to antithrombotic medication exposure are presented in Table B in S1 Appendix. As seen in Fig 1F, fatal outcome within 90 days following ICH was associated with use of warfarin plus aspirin and clopidogrel (HR 2.89; 95% CI, 1.49–5.60), warfarin plus aspirin (HR 1.37; 95% CI 1.11–1.68), aspirin and clopidogrel (HR 1.30; 95% CI, 1.05–1.61), and warfarin (HR 1.19; 95% CI, 1.09–1.31).

### Post-hoc analyses

Analyses with imputations from Statistics Norway had limited impact on the results (Table C and Figure B in S1 Appendix).

## Discussion

This nationwide study provides real-world data on risks of ICH in users of oral antithrombotic medications. Most antithrombotic drugs were associated with a significantly increased risk of ICH, with the highest incidence rates in users of warfarin plus aspirin and clopidogrel, warfarin plus aspirin, rivaroxaban plus aspirin, and aspirin-dypiridamole plus clopidogrel. Use of warfarin, warfarin plus aspirin, warfarin plus aspirin and clopidogrel (triple therapy), and aspirin plus clopidogrel was associated with higher risk of fatal outcome within 90 days following ICH. Increased one-year mortality was observed in users of antithrombotic medications for all ICH subgroups.

RCTs have established the relative efficacy or non-inferiority of the new oral anticoagulants (NOAC) dabigatran, apixaban and rivaroxaban compared with warfarin for the prevention of stroke or systemic embolism in patients with atrial fibrillation [14–16]. The observed risks of ICH are generally higher in the present real life setting than reported in RCTs. However, comparing results across other studies is challenging due to differences in reporting patterns of ICH. In a US insurance claims database study these three NOACs were associated with a lower risk of ICH in patients with atrial fibrillation compared to warfarin, with crude ICH rates per 100 person-years of 0.29 for apixaban, 0.28 for dabigatran, 0.44 for rivaroxaban and 0.79–1.06 for warfarin [7]. In contrast, we observed similar risks of ICH in users of apixaban, rivaroxaban, and warfarin. The incidence rate for hemorrhagic stroke in users of dabigatran in our study was similar to what was reported in an RCT comparing warfarin and dabigatran (0.10 to 0.12% per year) in patients with atrial fibrillation [14], and supports the findings from a Medicare claims database study reporting that use of dabigatran is associated with lower risk of ICH compared to warfarin [4]. Still, the low ICH risk in dabigatran users presented in our study might be attributed to limited observation time and insufficiently adjusted patient characteristics, and further investigations are warranted. Our results also differ from the findings of an RCT comparing apixaban and warfarin in patients with atrial fibrillation that found lower rates of ICH in the apixaban group (0.3% vs 0.8%) [15]. A trial of rivaroxaban versus warfarin in patients with atrial fibrillation reported a similar rate of ICH for rivaroxaban (0.5%) when compared to our study, but found higher incidence rates for warfarin (0.7%) [16]. Although we observed similar incidence rates of ICH for several anticoagulants, warfarin was the only one associated with increased risk of fatal outcome following ICH either as monotherapy, in combination with aspirin, or as part of triple therapy (Fig 1F). As demonstrated in Fig 2, one-year survival was significantly shorter in users of antithrombotic medication in all ICH subgroups. During the study period there were no NOAC reversal agents available, and the recent introduction of such agents may potentially influence case-fatality rates of ICH in NOAC users [17, 18].

Management of patients with an indication for oral anticoagulation who also have an indication for antiplatelet treatment due to intercurrent coronary disease is controversial with unclear treatment guidelines [19–22]. We present ICH risk for several combined anticoagulation and antiplatelet regimens, including NOACs plus aspirin, which are commonly used but have not been investigated in clinical trials. The risks of ICH for rivaroxaban plus aspirin were similar to warfarin plus aspirin, whereas dabigatran plus aspirin was not associated with increased risk. A Danish nationwide study in patients with myocardial infarction found a close to fourfold risk of hospital admission for bleeding complications with warfarin plus clopidogrel or triple therapy when compared to aspirin alone [5]. Our study shows a strong association between adjusted ICH hazard rates and combined antiplatelet and anticoagulation treatment, apart from dabigatran plus aspirin. We also found a higher incidence rate of ICH in patients on triple therapy than a retrospective study on patients with acute coronary syndrome [23]. However, one trial reported an even higher ICH rate of 1.1% for patients with myocardial infarction who underwent coronary stenting and were assigned to triple therapy [24].

Although aspirin has been available for more than a century, assessments of its benefits and risks are still challenging and inappropriate use for primary prevention of thrombotic disease is common [25, 26]. In a retrospective case-control study including 3,137 patients with either hemorrhagic stroke or subarachnoid hemorrhage, aspirin did not increase the risk of hemorrhagic stroke and decreased the risk of subarachnoid hemorrhage [27]. Further, warfarin use was associated with a greatly increased risk of hemorrhagic stroke and a moderately increased risk of subarachnoid hemorrhage. In our study, aspirin was associated with a minor increase in risk of hemorrhagic stroke without affecting the risk of subarachnoid hemorrhage (Fig 1B and 1D). However, we observed similar elevated risks of both hemorrhagic stroke and subarachnoid hemorrhage in warfarin users. Trials of dual antiplatelet treatment with aspirin plus clopidogrel have shown greater risks of life-threatening bleeding as compared with monotherapy [28, 29]. We also observed higher ICH risks for combinations of antiplatelet drugs compared to monotherapy. The ESPS-2 and ESPRIT trials provided evidence that the combination aspirin-dipyridamole was more effective in recurrent stroke prevention than either agent prescribed alone [30, 31]. In contrast to our findings, both trials found similar risk of major hemorrhage for aspirin-dipyridamole as compared with aspirin monotherapy. Later, the PRoFESS trial reported an increased risk of ICH among stroke patients treated with aspirin-dipyridamole (1.4%) as compared to patients treated with clopidogrel alone (1.0%), without finding evidence that either of the two treatments were superior to the other in recurrent stroke prevention [32].

Subdural hemorrhage, subarachnoid hemorrhage, and traumatic intracranial hemorrhage are often omitted from studies evaluating the efficacy and safety of antithrombotic medications despite the fact that the risks are clearly increased [33]. Traumatic intracranial hemorrhage accounted for 28% of ICH hospital admissions in our study with higher mortality in users of antithrombotic medications (Fig 1E). A population-based study found that antithrombotic medication increased the risk of being admitted to hospital with an injury, and strongly suggested that antithrombotic medication may increase physical damage following trauma [34]. Bleeding is a leading cause of preventable death following trauma [35], and future evaluations of antithrombotic medication should include traumatic intracranial hemorrhage.

Patients who survive ICH may have risk factors for future thromboembolic events, but the role of antithrombotic medications remains a therapeutic dilemma with conflicting evidence and contradictory recommendations [36–41]. There is currently a lack of solid evidence to guide decisions on whether and when to start or restart treatment in ICH survivors, and both well designed randomized trials and observational studies should be encouraged [42].

### Limitations

This study provides real-world data on risk of ICH in users of antithrombotic medications, and is an important adjunct to post-marketing surveillance based on spontaneously reported bleeding complications [43, 44].

There was a lack of information about important clinical parameters including body mass index, blood pressure values, tobacco use, lipid levels, and coagulation profile; hence a significant effect of unmeasured confounders cannot be excluded. Antithrombotic medication exposure is undeniably associated with medical conditions that affect frailty and the tendency to experience ICH. A bias toward overestimation of the true association can be tempered by confounding in the opposite direction because the association between antithrombotic medications and medical conditions is not monotonic. In very frail patients, the risk of ICH may increase disproportionately to the risk of thrombosis, and antithrombotic medication may be better avoided [45]. Tools are available to estimate the risk of bleeding for patients on anticoagulation treatment to help determine risk-benefit [46], but we had insufficient data on the necessary variables in our population.

Certain assumptions concerning drug exposure were made, and it should be emphasized that the DDD does not necessarily reflect the recommended or prescribed daily dose. Doses for individual patients and patient groups will often differ from the DDD and will necessarily have to be based on individual characteristics and pharmacokinetic considerations. Others have calculated exposure for each individual by estimating a daily dose after comparing the accumulated drug dose and the elapsed time from consecutive prescriptions for the drugs under investigation [47]. Information about the prescribed daily dose was unavailable in NorPD. However, the prescribed daily dose does not necessarily reflect actual drug doses consumed. There was likely to be some missing drug exposure in the first months of the study period for prescriptions dispensed in 2007. This might have increased the observed incidence rate of ICH in non-users of antithrombotic medication. Prescriptions dispensed in the last few months of 2014 might have extended beyond our study period, and some patients might have had an undetected ICH in early 2015 with ongoing exposure. Only ICHs resulting in hospital admission were included in the present study. These factors contribute to more conservative risk and incidence rate estimates of ICH in users of antithrombotic medications.

## Conclusion

The observed risks of ICH were higher than reported in RCTs, showing that there is still room for improvement in terms of antithrombotic medication safety. Warfarin plus aspirin and clopidogrel, warfarin plus aspirin, rivaroxaban plus aspirin, and aspirin-dypiridamole plus clopidogrel were associated with the highest risks of ICH. Use of warfarin, warfarin plus aspirin, warfarin plus aspirin and clopidogrel, and aspirin plus clopidogrel was associated with higher risk of fatal outcome within 90 days following ICH. In users of antithrombotic medications, increased one-year mortality was observed in all ICH subgroups.

## Supporting information

S1 Appendix Appendix. (DOCX)

S1 Checklist STROBE checklist. (DOC)

S1 Protocol Study protocol. (PDF)

## Abbreviations

ATC: Anatomical Therapeutic Chemical
DDD: Defined Daily Dose
ICD-10: 10^th^ revision of the International Statistical Classification of Diseases and Related Health Problems
ICH: Intracranial hemorrhage
NOAC: New oral anticoagulant
NorPD: Norwegian prescription database
NPR: Norwegian patient registry
RCT: Randomized controlled trial

## References

[1] Gonzalez-Perez A, Gaist D, Wallander MA, McFeat G, Garcia-Rodriguez LA. Mortality after hemorrhagic stroke: data from general practice (The Health Improvement Network). Neurology. 2013;81(6):559–65. Epub 2013/07/12. 10.1212/WNL.0b013e31829e6eff . 23843467

[2] Purrucker JC, Haas K, Rizos T, et al Early Clinical and Radiological Course, Management, and Outcome of Intracerebral Hemorrhage Related to New Oral Anticoagulants. JAMA Neurol. 2015 10.1001/jamaneurol.2015.3682 26660118

[3] Rothwell PM. Factors that can affect the external validity of randomised controlled trials. PLoS Clin Trials. 2006;1(1):e9 Epub 2006/07/28. 10.1371/journal.pctr.0010009 . 16871331 PMC1488890

[4] Hernandez I, Baik SH, Pinera A, Zhang Y. Risk of bleeding with dabigatran in atrial fibrillation. JAMA Intern Med. 2015;175(1):18–24. Epub 2014/11/05. 10.1001/jamainternmed.2014.5398 . 25365537 PMC6608584

[5] Sorensen R, Hansen ML, Abildstrom SZ, Hvelplund A, Andersson C, Jorgensen C, et al Risk of bleeding in patients with acute myocardial infarction treated with different combinations of aspirin, clopidogrel, and vitamin K antagonists in Denmark: a retrospective analysis of nationwide registry data. Lancet. 2009;374(9706):1967–74. Epub 2009/12/17. 10.1016/S0140-6736(09)61751-7 . 20006130

[6] Zapata-Wainberg G, Quintas S, Ximenez-Carrillo Rico A, Masjuan Vallejo J, Cardona P, Castellanos Rodrigo M, et al Epidemiology of Intracranial Hemorrhage Associated with Oral Anticoagulants in Spain: Trends in Anticoagulation Complications Registry—The TAC 2 Study. Interventional neurology. 2018;7(5):284–95. Epub 2018/05/17. 10.1159/000487518 . 29765398 PMC5939649

[7] Yao X, Abraham NS, Sangaralingham LR, Bellolio MF, McBane RD, Shah ND, et al Effectiveness and Safety of Dabigatran, Rivaroxaban, and Apixaban Versus Warfarin in Nonvalvular Atrial Fibrillation. J Am Heart Assoc. 2016;5(6). Epub 2016/07/15. 10.1161/jaha.116.003725 . 27412905 PMC4937291

[8] Gulati S, Solheim O, Carlsen SM, Oie LR, Jensberg H, Gulati AM, et al Risk of intracranial hemorrhage in users of oral antithrombotic drugs: Study protocol for a nationwide study. F1000Research. 2015;4:1519 Epub 2016/02/27. 10.12688/f1000research.7633.1 . 26918124 PMC4755390

[9] Norwegian Institute of Public Health. Over the counter medications in Norway 2017. https://www.fhi.no/hn/legemiddelbruk/omsetning-utenom-apotek/stabilt-butikksalg-av-reseptfrie-legemidler/.

[10] Varmdal T, Bakken IJ, Janszky I, Wethal T, Ellekjaer H, Rohweder G, et al Comparison of the validity of stroke diagnoses in a medical quality register and an administrative health register. Scand J Public Health. 2016;44(2):143–9. Epub 2015/12/15. 10.1177/1403494815621641 . 26660300

[11] Oie LR, Madsbu MA, Giannadakis C, Vorhaug A, Jensberg H, Salvesen O, et al Validation of intracranial hemorrhage in the Norwegian Patient Registry. Brain Behav. 2018;8(2):e00900 Epub 2018/02/28. 10.1002/brb3.900 . 29484261 PMC5822577

[12] Poulsen FR, Halle B, Pottegard A, Garcia Rodriguez LA, Hallas J, Gaist D. Subdural hematoma cases identified through a Danish patient register: diagnosis validity, clinical characteristics, and preadmission antithrombotic drug use. Pharmacoepidemiology and drug safety. 2016;25(11):1253–62. Epub 2016/11/03. 10.1002/pds.4058 . 27384945

[13] Guidelines for ATC classification and DDD assignment 2015. WHO Collaborating Centre for Drug Statistics Methodology. Oslo, 2014.

[14] Connolly SJ, Ezekowitz MD, Yusuf S, Eikelboom J, Oldgren J, Parekh A, et al Dabigatran versus warfarin in patients with atrial fibrillation. N Engl J Med. 2009;361(12):1139–51. Epub 2009/09/01. 10.1056/NEJMoa0905561 . 19717844

[15] Granger CB, Alexander JH, McMurray JJ, Lopes RD, Hylek EM, Hanna M, et al Apixaban versus warfarin in patients with atrial fibrillation. N Engl J Med. 2011;365(11):981–92. Epub 2011/08/30. 10.1056/NEJMoa1107039 . 21870978

[16] Patel MR, Mahaffey KW, Garg J, Pan G, Singer DE, Hacke W, et al Rivaroxaban versus warfarin in nonvalvular atrial fibrillation. N Engl J Med. 2011;365(10):883–91. Epub 2011/08/13. 10.1056/NEJMoa1009638 . 21830957

[17] Pollack CV Jr., Reilly PA, Eikelboom J, Glund S, Verhamme P, Bernstein RA, et al Idarucizumab for Dabigatran Reversal. N Engl J Med. 2015;373(6):511–20. Epub 2015/06/23. 10.1056/NEJMoa1502000 . 26095746

[18] Connolly SJ, Milling TJ Jr., Eikelboom JW, Gibson CM, Curnutte JT, Gold A, et al Andexanet Alfa for Acute Major Bleeding Associated with Factor Xa Inhibitors. N Engl J Med. 2016 Epub 2016/08/31. 10.1056/NEJMoa1607887 . 27573206 PMC5568772

[19] Anderson JL, Adams CD, Antman EM, Bridges CR, Califf RM, Casey DE Jr., et al ACC/AHA 2007 guidelines for the management of patients with unstable angina/non ST-elevation myocardial infarction: a report of the American College of Cardiology/American Heart Association Task Force on Practice Guidelines (Writing Committee to Revise the 2002 Guidelines for the Management of Patients With Unstable Angina/Non ST-Elevation Myocardial Infarction): developed in collaboration with the American College of Emergency Physicians, the Society for Cardiovascular Angiography and Interventions, and the Society of Thoracic Surgeons: endorsed by the American Association of Cardiovascular and Pulmonary Rehabilitation and the Society for Academic Emergency Medicine. Circulation. 2007;116(7):e148–304. Epub 2007/08/08. 10.1161/CIRCULATIONAHA.107.181940 . 17679616

[20] Bassand JP, Hamm CW, Ardissino D, Boersma E, Budaj A, Fernandez-Aviles F, et al Guidelines for the diagnosis and treatment of non-ST-segment elevation acute coronary syndromes. Eur Heart J. 2007;28(13):1598–660. Epub 2007/06/16. 10.1093/eurheartj/ehm161 . 17569677

[21] Fuster V, Ryden LE, Cannom DS, Crijns HJ, Curtis AB, Ellenbogen KA, et al ACC/AHA/ESC 2006 Guidelines for the Management of Patients with Atrial Fibrillation: a report of the American College of Cardiology/American Heart Association Task Force on Practice Guidelines and the European Society of Cardiology Committee for Practice Guidelines (Writing Committee to Revise the 2001 Guidelines for the Management of Patients With Atrial Fibrillation): developed in collaboration with the European Heart Rhythm Association and the Heart Rhythm Society. Circulation. 2006;114(7):e257–354. Epub 2006/08/16. 10.1161/CIRCULATIONAHA.106.177292 . 16908781

[22] Van de Werf F, Bax J, Betriu A, Blomstrom-Lundqvist C, Crea F, Falk V, et al Management of acute myocardial infarction in patients presenting with persistent ST-segment elevation: the Task Force on the Management of ST-Segment Elevation Acute Myocardial Infarction of the European Society of Cardiology. Eur Heart J. 2008;29(23):2909–45. Epub 2008/11/14. 10.1093/eurheartj/ehn416 . 19004841

[23] Braun OO, Bico B, Chaudhry U, Wagner H, Koul S, Tyden P, et al Concomitant use of warfarin and ticagrelor as an alternative to triple antithrombotic therapy after an acute coronary syndrome. Thromb Res. 2015;135(1):26–30. Epub 2014/12/04. 10.1016/j.thromres.2014.10.016 . 25467434

[24] Dewilde WJ, Oirbans T, Verheugt FW, Kelder JC, De Smet BJ, Herrman JP, et al Use of clopidogrel with or without aspirin in patients taking oral anticoagulant therapy and undergoing percutaneous coronary intervention: an open-label, randomised, controlled trial. Lancet. 2013;381(9872):1107–15. Epub 2013/02/19. 10.1016/S0140-6736(12)62177-1 . 23415013

[25] Mainous AG, Tanner RJ, Shorr RI, Limacher MC. Use of aspirin for primary and secondary cardiovascular disease prevention in the United States, 2011–2012. Journal of the American Heart Association. 2014;3(4). Epub 2014/07/16. 10.1161/jaha.114.000989 . 25023071 PMC4310388

[26] Mora S, Ames JM, Manson JE. Low-Dose Aspirin in the Primary Prevention of Cardiovascular Disease: Shared Decision Making in Clinical Practice. JAMA: the journal of the American Medical Association. 2016;316(7):709–10. Epub 2016/06/21. 10.1001/jama.2016.8362 . 27323335

[27] Garcia-Rodriguez LA, Gaist D, Morton J, Cookson C, Gonzalez-Perez A. Antithrombotic drugs and risk of hemorrhagic stroke in the general population. Neurology. 2013;81(6):566–74. Epub 2013/07/12. 10.1212/WNL.0b013e31829e6ffa . 23843464

[28] Bhatt DL, Fox KA, Hacke W, Berger PB, Black HR, Boden WE, et al Clopidogrel and aspirin versus aspirin alone for the prevention of atherothrombotic events. N Engl J Med. 2006;354(16):1706–17. Epub 2006/03/15. 10.1056/NEJMoa060989 . 16531616

[29] Diener HC, Bogousslavsky J, Brass LM, Cimminiello C, Csiba L, Kaste M, et al Aspirin and clopidogrel compared with clopidogrel alone after recent ischaemic stroke or transient ischaemic attack in high-risk patients (MATCH): randomised, double-blind, placebo-controlled trial. Lancet. 2004;364(9431):331–7. Epub 2004/07/28. . 15276392 10.1016/S0140-6736(04)16721-4

[30] Diener HC, Cunha L, Forbes C, Sivenius J, Smets P, Lowenthal A. European Stroke Prevention Study. 2. Dipyridamole and acetylsalicylic acid in the secondary prevention of stroke. J Neurol Sci. 1996;143(1–2):1–13. Epub 1996/11/01. . 8981292 10.1016/s0022-510x(96)00308-5

[31] Halkes PH, van Gijn J, Kappelle LJ, Koudstaal PJ, Algra A. Aspirin plus dipyridamole versus aspirin alone after cerebral ischaemia of arterial origin (ESPRIT): randomised controlled trial. Lancet. 2006;367(9523):1665–73. Epub 2006/05/23. 10.1016/S0140-6736(06)68734-5 . 16714187

[32] Sacco RL, Diener HC, Yusuf S, Cotton D, Ounpuu S, Lawton WA, et al Aspirin and extended-release dipyridamole versus clopidogrel for recurrent stroke. N Engl J Med. 2008;359(12):1238–51. Epub 2008/08/30. 10.1056/NEJMoa0805002 . 18753638 PMC2714259

[33] Gaist D, Garcia Rodriguez LA, Hellfritzsch M, Poulsen FR, Halle B, Hallas J, et al Association of Antithrombotic Drug Use With Subdural Hematoma Risk. JAMA: the journal of the American Medical Association. 2017;317(8):836–46. Epub 2017/03/01. 10.1001/jama.2017.0639 . 28245322

[34] Di Bartolomeo S, Marino M, Valent F, De Palma R. Effects of anticoagulant and antiplatelet drugs on the risk for hospital admission for traumatic injuries: a case-control and population-based study. The journal of trauma and acute care surgery. 2014;76(2):437–42. Epub 2014/01/09. 10.1097/TA.0b013e3182aa80f9 . 24398774

[35] Pfeifer R, Tarkin IS, Rocos B, Pape HC. Patterns of mortality and causes of death in polytrauma patients—has anything changed? Injury. 2009;40(9):907–11. Epub 2009/06/23. 10.1016/j.injury.2009.05.006 . 19540488

[36] Pennlert J, Asplund K, Carlberg B, Wiklund PG, Wisten A, Asberg S, et al Antithrombotic Treatment Following Intracerebral Hemorrhage in Patients With and Without Atrial Fibrillation. Stroke. 2015;46(8):2094–9. Epub 2015/07/15. 10.1161/STROKEAHA.115.009087 . 26159794

[37] Morgenstern LB, Hemphill JC 3rd, Anderson C, Becker K, Broderick JP, Connolly ES Jr., et al Guidelines for the management of spontaneous intracerebral hemorrhage: a guideline for healthcare professionals from the American Heart Association/American Stroke Association. Stroke. 2010;41(9):2108–29. Epub 2010/07/24. 10.1161/STR.0b013e3181ec611b . 20651276 PMC4462131

[38] Steiner T, Al-Shahi Salman R, Beer R, Christensen H, Cordonnier C, Csiba L, et al European Stroke Organisation (ESO) guidelines for the management of spontaneous intracerebral hemorrhage. Int J Stroke. 2014;9(7):840–55. Epub 2014/08/27. 10.1111/ijs.12309 . 25156220

[39] Flynn RW, MacDonald TM, Murray GD, Doney AS. Systematic review of observational research studying the long-term use of antithrombotic medicines following intracerebral hemorrhage. Cardiovasc Ther. 2010;28(3):177–84. Epub 2010/03/27. 10.1111/j.1755-5922.2009.00118.x . 20337638

[40] Biffi A, Kuramatsu JB, Leasure A, Kamel H, Kourkoulis C, Schwab K, et al Oral Anticoagulation and Functional Outcome after Intracerebral Hemorrhage. Ann Neurol. 2017;82(5):755–65. Epub 2017/10/14. 10.1002/ana.25079 . 29028130 PMC5730065

[41] Gonzalez-Perez A, Gaist D, de Abajo FJ, Saez ME, Garcia Rodriguez LA. Low-Dose Aspirin after an Episode of Haemorrhagic Stroke Is Associated with Improved Survival. Thromb Haemost. 2017;117(12):2396–405. Epub 2017/12/07. 10.1160/TH17-05-0342 . 29212127

[42] Perry LA, Berge E, Bowditch J, Forfang E, Ronning OM, Hankey GJ, et al Antithrombotic treatment after stroke due to intracerebral haemorrhage. The Cochrane database of systematic reviews. 2017;5:Cd012144 Epub 2017/05/26. 10.1002/14651858.CD012144.pub2 . 28540976 PMC6481874

[43] Navgren M, Forsblad J, Wieloch M. Bleeding complications related to warfarin treatment: a descriptive register study from the anticoagulation clinic at Helsingborg Hospital. J Thromb Thrombolysis. 2013 Epub 2013/11/19. 10.1007/s11239-013-1011-z . 24242025

[44] Southworth MR, Reichman ME, Unger EF. Dabigatran and postmarketing reports of bleeding. N Engl J Med. 2013;368(14):1272–4. Epub 2013/03/15. 10.1056/NEJMp1302834 . 23484796

[45] Camm AJ, Kirchhof P, Lip GY, Schotten U, Savelieva I, Ernst S, et al Guidelines for the management of atrial fibrillation: the Task Force for the Management of Atrial Fibrillation of the European Society of Cardiology (ESC). European heart journal. 2010;31(19):2369–429. Epub 2010/08/31. 10.1093/eurheartj/ehq278 . 20802247

[46] Pisters R, Lane DA, Nieuwlaat R, de Vos CB, Crijns HJ, Lip GY. A novel user-friendly score (HAS-BLED) to assess 1-year risk of major bleeding in patients with atrial fibrillation: the Euro Heart Survey. Chest. 2010;138(5):1093–100. Epub 2010/03/20. 10.1378/chest.10-0134 . 20299623

[47] Schjerning Olsen AM, Gislason GH, McGettigan P, Fosbol E, Sorensen R, Hansen ML, et al Association of NSAID use with risk of bleeding and cardiovascular events in patients receiving antithrombotic therapy after myocardial infarction. JAMA. 2015;313(8):805–14. Epub 2015/02/25. 10.1001/jama.2015.0809 . 25710657

