# Supplementary material for: Risk of intracranial hemorrhage (RICH) in users of oral antithrombotic drugs: Nationwide pharmacoepidemiological study
Source: PLoS One. 2018 Aug 23;13(8):e0202575. doi: 10.1371/journal.pone.0202575 (PMC6107180; doi:10.1371/journal.pone.0202575)
Supplement: S1 Appendix — (DOCX) [file pone.0202575.s001.docx]

**Risk of intracranial hemorrhage in users of oral antithrombotic drugs: A nationwide registry-based pharmacoepidemiological study**

**Supplementary appendix**

**Method A**

Comorbidities and accidents were identified from both NPR and NorPD, including atrial fibrillation (ICD-10: I48; ICPC-2: K78), congestive heart failure (ICD-10: I11.0, I42, I50, J81; ICPC-2: K77, K82, K83, K84), heart valve disease (ICD-10: I05-I08, I34-I37, Q22-Q23), thromboembolism (ICD-10: I63–I68, I20–125, I74, G45.8, G45.9; ICPC-2: K93, K94), vascular disease (ICD-10: I21, I22, I70.0, I70.2–I70.9, F01; ICPC-2: K74-K76, K89-K92, K99), hypertension (ICD-10: I10–I15; ICPC-2: K85-K87), diabetes mellitus (ICD-10: E10–E14; ICPC-2: T89, T90), peptic ulcer (ICD-10: K25–K29), liver disease (ICD-10: B15–19, C22, D68.4, K70–K77, Z94.4), alcohol abuse (ICD-10: G31.2, G62.1, G72.1, I42.6, K29.2, K70, K86.0, O35.4, T51, Z71.4, Z72.1), osteoarthritis (ICD-10: M19), extracranial bleeding (ICD-10: I69.0–I69.2, J94.2, K25.0, K25.4, K26.0, K26.4, K27.0, K28.0, K92.0–K92.2, N02, R04, R31), and chronic renal failure (ICD-10: E10.2, E11.2, E13.2, E14.2, I12.0, N00–N08, N11, N12, N14, N17–N19, N26, N15.8–N16.0, N16.2–N16.4, Q61), and accidents (ICD-10: V0n–Y3n, S06.0–S06.9, S10–T19).

**Results A**

Figure A. Flow diagram with study enrolment

3,131,270 patients ≥18 years in the Norwegian Patient Registry and/or the Norwegian Prescription Database

6,279 with traumatic intracranial hemorrhage

4,487 with subdural

hemorrhage

2,680 with subarachnoid hemorrhage

8,665 with hemorrhagic stroke

Table A. Characteristics of the study population according to OAM exposure

22,111 with intracranial hemorrhage

| **Exposure Group** | **Total Population** | **Any antithrombotic treatment** | **Aspirin** | **Warfarin** | **Aspirin plus Clopidogrel** | **Aspirin-Dipyridamole** | **Warfarin plus Aspirin** |
| --- | --- | --- | --- | --- | --- | --- | --- |
| Patients, No. | 3131270 | 729818 | 594761 | 151966 | 83593 | 59698 | 54152 |
| Age, mean (SD), y | 48.5 (19.1) | 66.3 (14.2) | 66.1 (14.0) | 69.5 (14.2) | 65.0 (12.6) | 68.4 (12.6) | 70.7 (11.3) |
| Women, No. (%) | 1669209 (53.3) | 334278 (45.8) | 274717 (46.2) | 63628 (41.9) | 27302 (32.7) | 26760 (44.8) | 20393 (37.7) |
| **Comorbidity** |  |  |  |  |  |  |  |
| Atrial fibrillation | 227986 (7.3) | 195143 (26.7) | 117665 (19.8) | 112672 (74.1) | 19579 (23.4) | 10367 (17.4) | 44851 (82.8) |
| Thromboembolism | 364978 (11.7) | 325392 (44.6) | 264963 (44.5) | 75739 (49.8) | 79593 (95.2) | 46398 (77.7) | 36290 (67.0) |
| Vascular disease | 339561 (10.8) | 298149 (40.9) | 238983 (40.2) | 71936 (47.3) | 68264 (81.7) | 52216 (87.5) | 32865 (60.7) |
| Heart valve disease | 99240 (3.2) | 74517 (10.2) | 54344 (9.1) | 30497 (20.1) | 11247 (13.4) | 5626 (9.4) | 13589 (25.1) |
| Congestive heart failure | 864277 (27.6) | 491134 (67.3) | 406438 (68.3) | 107714 (70.9) | 63290 (75.7) | 41692 (69.8) | 44960 (83.0) |
| Alcohol abuse | 13492 (0.4) | 4213 (0.6) | 3446 (0.6) | 789 (0.5) | 537 (0.6) | 461 (0.8) | 271 (0.5) |
| Liver disease | 45837 (1.5) | 12762 (1.8) | 10029 (1.7) | 3351 (2.2) | 1603 (1.9) | 994 (1.7) | 1203 (2.2) |
| Osteoarthritis | 56778 (1.8) | 18419 (2.5) | 15146 (2.5) | 3739 (2.5) | 2271 (2.7) | 1544 (2.6) | 1521 (2.8) |
| Peptic ulcer | 96615 (3.1) | 45012 (6.2) | 36984 (6.2) | 10645 (7.0) | 7610 (9.1) | 4421 (7.4) | 4618 (8.5) |
| Diabetes mellitus | 228348 (7.3) | 129191 (17.7) | 108981 (18.3) | 26237 (17.3) | 18087 (21.6) | 11067 (18.5) | 11686 (21.6) |
| Hypertension | 388444 (12.4) | 260130 (35.6) | 214753 (36.1) | 60720 (40.0) | 43567 (52.1) | 30457 (51.0) | 28940 (53.4) |
| Chronic renal failure | 125140 (4.0) | 81733 (11.2) | 63673 (10.7) | 26480 (17.4) | 12824 (15.3) | 7944 (13.3) | 11476 (21.2) |
| Previous bleeding | 148506 (4.7) | 76886 (10.5) | 60016 (10.1) | 23301 (15.3) | 11279 (13.5) | 7362 (12.3) | 9470 (17.5) |
| **Concomitant medication** |  |  |  |  |  |  |  |
| NSAID | 1954797 (62.4) | 299475 (41.0) | 245880 (41.3) | 32327 (21.3) | 14993 (17.9) | 17248 (28.9) | 6534 (12.1) |
| Statin | 676318 (21.6) | 456416 (62.5) | 388922 (65.4) | 67941 (44.7) | 76578 (91.6) | 47998 (80.4) | 34665 (64.0) |
| Thiazide | 708589 (22.6) | 395137 (54.1) | 319946 (53.8) | 81393 (53.6) | 50435 (60.3) | 31338 (52.5) | 33448 (61.8) |
| Loop diuretic | 305002 (9.7) | 188503 (25.8) | 129733 (21.8) | 60803 (40.0) | 19460 (23.3) | 10461 (17.5) | 20909 (38.6) |
| Renin-angiotensin system inhibitor | 123060 (3.9) | 49867 (6.8) | 40409 (6.8) | 8345 (5.5) | 3570 (4.3) | 3631 (6.1) | 2645 (4.9) |
| Antiarrhythmic drug | 762272 (24.3) | 471951 (64.7) | 369493 (62.1) | 111507 (73.4) | 67678 (81.0) | 30657 (51.4) | 45053 (83.2) |
| Proton-pump inhibitor | 735645 (23.5) | 220805 (30.2) | 171205 (28.8) | 37618 (24.8) | 25352 (30.3) | 16668 (27.9) | 13445 (24.8) |
| Glucose-lowering drug | 212442 (6.8) | 113495 (15.6) | 93917 (15.8) | 20902 (13.8) | 13226 (15.8) | 9049 (15.2) | 8478 (15.7) |
| Antidepressants | 588669 (18.8) | 139623 (19.1) | 108895 (18.3) | 24259 (16.0) | 10796 (12.9) | 13033 (21.8) | 6530 (12.1) |
| Glucocorticoid | 609389 (19.5) | 154094 (21.1) | 113736 (19.1) | 31499 (20.7) | 10084 (12.1) | 9527 (16.0) | 7641 (14.1) |

Table A (continued, 2/3)

| **Exposure Group** | **Clopidogrel** | **Rivaroxaban** | **Dipyridamole** | **Dabigatran** | **Aspirin plus Ticagrelor** | **Apixaban** | **Warfarin plus Aspirin and Clopidogrel** | **Dabigatran plus Aspirin** | **Rivaroxaban plus Aspirin** |
| --- | --- | --- | --- | --- | --- | --- | --- | --- | --- |
| Patients, No. | 44771 | 23532 | 22329 | 18541 | 11896 | 8202 | 7682 | 6929 | 6740 |
| Age, mean (SD), y | 66.0 (12.6) | 64.4 (14.1) | 70.3 (12.1) | 66.9 (11.3) | 58.5 (11.3) | 66.3 (11.5) | 68.4 (10.9) | 68.5 (9.9) | 69.4 (10.3) |
| Women, No. (%) | 16034 (35.8) | 11000 (46.7) | 9914 (44.4) | 7784 (42.0) | 3068 (25.8) | 4148 (50.6) | 2012 (26.2) | 2686 (38.8) | 2959 (44.0) |
| **Comorbidity** |  |  |  |  |  |  |  |  |  |
| Atrial fibrillation | 10669 (23.8) | 14935 (63.5) | 4094 (18.3) | 16868 (91.0) | 1488 (12.5) | 5962 (72.7) | 6075 (79.1) | 6476 (93.5) | 5248 (77.9) |
| Thromboembolism | 39884 (89.1) | 10506 (44.7) | 15467 (69.3) | 6348 (34.2) | 11805 (99.2) | 2506 (30.6) | 7566 (98.5) | 3731 (53.9) | 4062 (60.3) |
| Vascular disease | 34884 (77.9) | 6908 (29.4) | 20283 (90.8) | 6060 (32.7) | 11306 (95.0) | 2166 (26.4) | 6790 (88.4) | 3206 (46.3) | 3183 (47.2) |
| Heart valve disease | 6194 (13.8) | 2664 (11.2) | 2338 (10.5) | 2671 (14.4) | 960 (8.1) | 1049 (12.8) | 2199 (28.6) | 1232 (17.8) | 1080 (16.0) |
| Congestive heart failure | 33785 (75.5) | 14389 (61.2) | 15991 (71.6) | 13457 (72.6) | 7742 (65.1) | 5478 (66.8) | 6785 (88.3) | 5637 (81.4) | 5339 (80.5) |
| Alcohol abuse | 371 (0.8) | 177 (0.8) | 189 (0.8) | 128 (0.7) | 63 (0.5) | 37 (0.5) | 43 (0.6) | 45 (0.7) | 48 (0.7) |
| Liver disease | 963 (2.1) | 456 (1.9) | 383 (1.7) | 317 (1.7) | 207 (1.7) | 96 (1.2) | 198 (2.6) | 114 (1.7) | 102 (1.5) |
| Osteoarthritis | 1268 (2.8) | 835 (3.6) | 602 (2.7) | 603 (3.3) | 352 (3.0) | 336 (4.1) | 211 (2.8) | 243(3.5) | 271 (4.0) |
| Peptic ulcer | 4992 (11.2) | 1503 (6.4) | 1855 (8.3) | 1168 (6.3) | 818 (6.9) | 496 (6.1) | 871 (11.3) | 495 (7.1) | 511 (7.6) |
| Diabetes mellitus | 9785 (21.9) | 2888 (12.3) | 4367 (19.6) | 2637 (14.2) | 2310 (19.4) | 1031 (12.6) | 2083 (27.1) | 1258 (18.2) | 1210 (18.0) |
| Hypertension | 23591 (52.7) | 8474 (36.0) | 10959 (49.1) | 7925 (42.7) | 5752 (48.4) | 3171 (38.7) | 4618 (60.1) | 3630 (52.4) | 3421 (50.8) |
| Chronic renal failure | 7417 (16.6) | 2210 (9.4) | 3370 (15.1) | 1624 (8.8) | 1091 (9.2) | 835 (10.2) | 1970 (25.6) | 740 (10.7) | 916 (13.6) |
| Previous bleeding | 6896 (15.4) | 2867 (12.2) | 3026 (13.6) | 2404 (13.0) | 1196 (10.1) | 876 (10.7) | 1711 (22.3) | 956 (13.8) | 1003 (14.9) |
| **Concomitant medication** |  |  |  |  |  |  |  |  |  |
| NSAID | 6698 (15.0) | 4000 (17.0) | 3087 (13.8) | 3199 (17.3) | 1892 (15.9) | 1767 (21.5) | 528 (6.9) | 863 (12.5) | 853 (12.7) |
| Statin | 36827 (82.3) | 8183 (34.8) | 15379 (68.9) | 7870 (42.5) | 11556 (97.1) | 3066 (37.4) | 6873 (89.5) | 4285 (61.8) | 4094 (60.7) |
| Thiazide | 24166 (54.0) | 10092 (42.9) | 10258 (45.9) | 9816 (52.9) | 7388 (62.1) | 3889 (47.4) | 5751 (74.9) | 4206 (60.7) | 3881 (57.6) |
| Loop diuretic | 9171 (20.5) | 4753 (20.2) | 3006 (13.5) | 4522 (24.4) | 1812 (15.2) | 1723 (21.0) | 3432 (44.7) | 1776 (25.6) | 1720 (25.5) |
| Renin-angiotensin system inhibitor | 1670 (3.7) | 692 (2.9) | 1102 (4.9) | 590 (3.2) | 295 (2.5) | 239 (2.9) | 313 (4.1) | 229 (3.3) | 238 (3.5) |
| Antiarrhythmic drug | 26519 (59.2) | 13205 (56.1) | 9690 (43.4) | 14598 (78.7) | 9990 (84.0) | 5264 (64.2) | 6885 (89.6) | 5741 (82.9) | 5063 (75.1) |
| Proton-pump inhibitor | 13070 (29.2) | 5216 (22.2) | 4702 (21.1) | 4448 (24.0) | 4056 (34.1) | 1723 (21.0) | 2707 (35.2) | 1689 (24.4) | 1729 (25.7) |
| Glucose-lowering drug | 6736 (15.1) | 2153 (9.2) | 2970 (13.3) | 2050 (11.1) | 1852 (15.6) | 740 (9.0) | 1489 (19.4) | 946 (13.7) | 906 (13.4) |
| Antidepressants | 6223 (13.9) | 2836 (12.91 | 3859 (17.3) | 2056 (11.1) | 1324 (11.1) | 863 (10.5) | 733 (9.5) | 685 (9.9) | 746 (11.1) |
| Glucocorticoid | 5590 (12.5) | 2965 (12.6) | 2266 (10.2) | 2281 (12.3) | 1197 (10.1) | 750 (9.1) | 840 (10.9) | 716 (10.3) | 721 (10.7) |

Table A (continued, 3/3)

| **Exposure Group** | **Aspirin-Dipyridamole plus Clopidogrel** | **Other oral antithrombotic treatment** |
| --- | --- | --- |
| Patients, No. | 5835 | 24734 |
| Age, mean (SD), y | 67.4 (12.0) | 65.6 (12.2) |
| Women, No. (%) | 2460 (42.2) | 8579 (34.7) |
| **Comorbidity** |  |  |
| Atrial fibrillation | 1103 (18.9) | 14012 (56.6) |
| Thromboembolism | 5507 (94.4) | 20370 (82.4) |
| Vascular disease | 5332 (91.4) | 19618 (79.3) |
| Heart valve disease | 778 (13.3) | 3987 (16.1) |
| Congestive heart failure | 4474 (76.7) | 10639 (78.2) |
| Alcohol abuse | 37 (0.6) | 156 (0.6) |
| Liver disease | 115 (2.0) | 492 (2.0) |
| Osteoarthritis | 176 (3.0) | 767 (3.1) |
| Peptic ulcer | 585 (10.0) | 2173 (8.8) |
| Diabetes mellitus | 1301 (22.3) | 5324 (21.5) |
| Hypertension | 3710 (63.6) | 13509 (54.6) |
| Heart failure | 4474 (76.7) | 19076 (77.1) |
| Chronic renal failure | 998 (17.1) | 3897 (15.8) |
| Previous bleeding | 910 (15.6) | 3855 (15.6) |
| **Concomitant medication** |  |  |
| NSAID | 544 (9.3) | 2729 (11.0) |
| Statin | 5164 (88.5) | 19626 (79.4) |
| Thiazide | 3246 (55.6) | 15213 (61.5) |
| Loop diuretic | 919 (15.8) | 6131 (24.8) |
| Renin-angiotensin system inhibitor | 264 (4.5) | 820 (3.3) |
| Antiarrhythmic drug | 3232 (55.4) | 18315 (74.1) |
| Proton-pump inhibitor | 1607 (27.5) | 7292 (29.5) |
| Glucose-lowering drug | 950 (16.3) | 3982 (16.1) |
| Antidepressants | 877 (15.0) | 2940 (11.9) |
| Glucocorticoid | 464 (8.0) | 2719 (11.0) |

Table B. Fatal outcome, defined as death within 90 days, following ICH by drug exposure group

| **Antithrombotic treatment** | **Intracranial hemorrhage, No.** | **Fatal outcome, No. (%, 95% CI)** |
| --- | --- | --- |
| None | 14056 | 3228 (23, 22-24) |
| Aspirin | 4701 | 1423 (30, 29-32) |
| Warfarin | 1678 | 645 (38, 36-41) |
| Aspirin plus Clopidogrel | 264 | 93 (35, 29-41) |
| Aspirin-Dipyridamole | 650 | 191 (29, 26-33) |
| Warfarin plus Aspirin | 263 | 99 (38, 32-44) |
| Clopidogrel | 162 | 39 (24, 18-31) |
| Rivaroxaban | 91 | 32 (35, 25-46) |
| Dipyridamole | 49 | 14 (29, 17-43) |
| Dabigatran | 40 | 12 (30, 17-47) |
| Ticagrelor plus Aspirin | 25 | 7 (28, 12-49) |
| Apixaban | 12 | 6 (50, 21-79) |
| Warfarin plus Aspirin and Clopidogrel | 17 | 9 (53, 28-77) |
| Dabigatran plus Aspirin | 10 | 2 (20, 2.5-56) |
| Rivaroxaban plus Aspirin | 20 | 6 (30, 12-54) |
| Aspirin-Dipyridamole plus Clopidogrel | 14 | 4 (29, 8.0-58) |
| Other antithrombotic medications | 59 | 21 (36, 24-49) |

Table C. Incidence rates for intracranial hemorrhage by drug exposure group including controls from Statistics Norway. One patient could have multiple treatment courses with one drug or with different drugs.

| **Antithrombotic treatment** | **Patients, No.** | **Patient-years at risk** | **Events, No.** | **Crude Rate, Events per 100 Person-Years (95% CI)** | **Sex- and age-adjusted incidence rate, Events per 100 Person-Years (95% CI)** |
| --- | --- | --- | --- | --- | --- |
| None | 3671014 | 22934700.5 | 14056 | 0.061 (0.060-0.062) | 0.075 (0.074-0.076) |
| Aspirin | 594761 | 1998423.3 | 4701 | 0.24 (0.23-0.24) | 0.11 (0.098-0.13) |
| Warfarin | 151966 | 306658.9 | 1678 | 0.55 (0.52-0.57) | 0.26 (0.22-0.29) |
| Aspirin plus Clopidogrel | 83593 | 72635.1 | 264 | 0.36 (0.32-0.41) | 0.18 (0.14-0.23) |
| Aspirin-Dipyridamole | 59698 | 131792.9 | 650 | 0.49 (0.46-0.53) | 0.28 (0.18-0.38) |
| Warfarin plus Aspirin | 54152 | 35035.2 | 263 | 0.75 (0.66-0.85) | 0.31 (0.23-0.40) |
| Clopidogrel | 44771 | 43010.8 | 162 | 0.38 (0.32-0.44) | 0.24 (0.14-0.33) |
| Rivaroxaban | 23532 | 17873.3 | 91 | 0.51 (0.41-0.63) | 0.24 (0.16-0.31) |
| Dipyridamole | 22329 | 10868.0 | 49 | 0.45 (0.33-0.60) | 0.14 (0.085-0.19) |
| Dabigatran | 18541 | 16176.3 | 40 | 0.25 (0.18-0.34) | 0.085 (0.047-0.12) |
| Ticagrelor plus Aspirin | 11896 | 8829.8 | 25 | 0.28 (0.18-0.42) | 0.18 (0.091-0.27) |
| Apixaban | 8202 | 2688.0 | 12 | 0.45 (0.23-0.78) | 0.14 (0.055-0.23) |
| Warfarin plus Aspirin and Clopidogrel | 7682 | 2008.7 | 17 | 0.85 (0.49-1.36) | 0.29 (0.061-0.53) |
| Dabigatran plus Aspirin | 6929 | 2487.3 | 10 | 0.40 (0.19-0.74) | 0.21 (0.019-0.41) |
| Rivaroxaban plus Aspirin | 6740 | 2211.9 | 20 | 0.90 (0.55-1.40) | 0.32 (0.13-0.51) |
| Aspirin-Dipyridamole plus Clopidogrel | 5835 | 1072.6 | 14 | 1.31 (0.71-2.19) | 0.39 (0.16-0.62) |
| Other antithrombotic medication | 24734 | 9986.2 | 59 | 0.59 (0.45-0.76) | 0.23 (0.16-0.29) |

Figure B. Risk of intracranial hemorrhage according to oral antithrombotic medication exposure including controls from Statistics Norway
