# Supplementary material for: Risk of intracranial hemorrhage (RICH) in users of oral antithrombotic drugs: Nationwide pharmacoepidemiological study
Source: PLoS One. 2018 Aug 23;13(8):e0202575. doi: 10.1371/journal.pone.0202575 (PMC6107180; doi:10.1371/journal.pone.0202575)
Supplement: S1 Checklist — (DOC) [file pone.0202575.s002.doc]

STROBE Statement—Checklist of items that should be included in reports of ***cohort studies***

|  | Item No | Recommendation |
| --- | --- | --- |
| **Title and abstract** | 1 | (*a*) Indicate the study’s design with a commonly used term in the title or the abstract **PAGE 1** |
|  |  | (*b*) Provide in the abstract an informative and balanced summary of what was done and what was found  **PAGES 4-5** |
| Introduction | | |
| Background/rationale | 2 | Explain the scientific background and rationale for the investigation being reported  **PAGE 6: “**Among hemorrhagic complications (…)” |
| Objectives | 3 | State specific objectives, including any prespecified hypotheses  **PAGE 6 “**The objective of this nationwide study (…)” |
| Methods | | |
| Study design | 4 | Present key elements of study design early in the paper  **PAGE 7 “Study population (…)”** |
| Setting | 5 | Describe the setting, locations, and relevant dates, including periods of recruitment, exposure, follow-up, and data collection  **PAGES 7-9 In the following sections: “***Study population”, “The Norwegian health care system”, “The Norwegian Prescription Database”, and “The Norwegian Patient Registry”.* |
| Participants | 6 | 1. Give the eligibility criteria, and the sources and methods of selection of participants. Describe methods of follow-up 2. **PAGES 7 Section: “***Study population”* |
|  |  | (*b*) For matched studies, give matching criteria and number of exposed and unexposed **N/A** |
| Variables | 7 | Clearly define all outcomes, exposures, predictors, potential confounders, and effect modifiers. Give diagnostic criteria, if applicable **PAGES 7-10 In the following sections:** “Outcome measures”, “The Norwegian Prescription Database”, and “The Norwegian Patient Registry”, “Assessment of exposure to antithrombotic medications”, “Potential confounders and risk factors” |
| Data sources/ measurement | 8* | For each variable of interest, give sources of data and details of methods of assessment (measurement). Describe comparability of assessment methods if there is more than one group **PAGES 7-9, PAGES 7-9 In the following sections:** “Study population”, “The Norwegian health care system”, “The Norwegian Prescription Database”,“The Norwegian Patient Registry”, and S1 Methods in the Appendix. |
| Bias | 9 | Describe any efforts to address potential sources of bias **PAGE 7-11 In the following sections:** “The Norwegian Prescription Database”, “The Norwegian Patient Registry”, “Potential confounders and risk factors”, and “Missing data (p 11)”. **PAGE 21:** “Limitations” |
| Study size | 10 | Explain how the study size was arrived at **PAGE 7:** “Study population”. This is a nationwide study. |
| Quantitative variables | 11 | Explain how quantitative variables were handled in the analyses. If applicable, describe which groupings were chosen and why **PAGES 10-11 (Statistics), PAGES 9 (Assessment of exposure to antithrombotic medications)** |
| Statistical methods | 12 | (*a*) Describe all statistical methods, including those used to control for confounding **PAGE 9-11 (“Assessment of exposure to antithrombotic medications”, “Potential confounders and risk factors”, “Statistics”, “Missing data”)** |
|  |  | (*b*) Describe any methods used to examine subgroups and interactions **PAGE 9-11 (“Statistics”)** |
|  |  | (*c*) Explain how missing data were addressed **PAGE 11 (“Missing data”)** |
|  |  | (*d*) If applicable, explain how loss to follow-up was addressed, **N/A** |
|  |  | (*e*) Describe any sensitivity analyses **PAGE 11 (“Missing data”)** |
| Results | | |
| Participants | 13* | (a) Report numbers of individuals at each stage of study—eg numbers potentially eligible, examined for eligibility, confirmed eligible, included in the study, completing follow-up, and analysed **PAGE 12 (Results -> Study population & S1 Figure in the Appendix** |
|  |  | (b) Give reasons for non-participation at each stage **PAGE 12 & S1 Figure in the Appendix** |
|  |  | (c) Consider use of a flow diagram **S1 Figure in the Appendix** |
| Descriptive data | 14* | (a) Give characteristics of study participants (eg demographic, clinical, social) and information on exposures and potential confounders **S1 Table in the Appendix** |
|  |  | (b) Indicate number of participants with missing data for each variable of interest **PAGE 18 (Post-hoc analyses) & S3 Table and S2 Figure in the Appendix** |
|  |  | (c) Summarise follow-up time (eg, average and total amount) **PAGE 15 (TABLE 1)** |
| Outcome data | 15* | Report numbers of outcome events or summary measures over time **PAGE 12 (Primary outcome),** **PAGE 15 (TABLE 1), PAGE 15-16 (TABLE 2)** |
| Main results | 16 | (*a*) Give unadjusted estimates and, if applicable, confounder-adjusted estimates and their precision (eg, 95% confidence interval). Make clear which confounders were adjusted for and why they were included **PAGE 11-12 (Primary outcome),** **PAGE 14 (TABLE 1), PAGE 16 (TABLE 2), Fig 1** |
|  |  | (*b*) Report category boundaries when continuous variables were categorized **PAGES 12 (Primary outcome), PAGE 15 (TABLE 1), PAGE 16 (TABLE 2), FIG 1 & FIG 2.** |
|  |  | (*c*) If relevant, consider translating estimates of relative risk into absolute risk for a meaningful time period **PAGE 15 (TABLE 1).** |
| Other analyses | 17 | Report other analyses done—eg analyses of subgroups and interactions, and sensitivity analyses **PAGE 18 (Post-hoc analyses) & S3 Table and S2 Figure in the Appendix** |
| Discussion | | |
| Key results | 18 | Summarise key results with reference to study objectives **PAGE 18 (First paragraph of the discussion).** |
| Limitations | 19 | Discuss limitations of the study, taking into account sources of potential bias or imprecision. Discuss both direction and magnitude of any potential bias. **PAGES 21-22 (Section: “Limitations”)** |
| Interpretation | 20 | Give a cautious overall interpretation of results considering objectives, limitations, multiplicity of analyses, results from similar studies, and other relevant evidence. **PAGE 18-21 (From the second paragraph in the discussion to the limitations section), PAGE 23 (Conclusion).** |
| Generalisability | 21 | Discuss the generalisability (external validity) of the study results. **PAGE 21 “**This study provides real-world data on risk of ICH (…)” |
| Other information | | |
| Funding | 22 | Give the source of funding and the role of the funders for the present study and, if applicable, for the original study on which the present article is based. **PAGE 24 (Section: “Funding”).** |

*Give information separately for exposed and unexposed groups.

**Note:** An Explanation and Elaboration article discusses each checklist item and gives methodological background and published examples of transparent reporting. The STROBE checklist is best used in conjunction with this article (freely available on the Web sites of PLoS Medicine at http://www.plosmedicine.org/, Annals of Internal Medicine at http://www.annals.org/, and Epidemiology at http://www.epidem.com/). Information on the STROBE Initiative is available at http://www.strobe-statement.org.
